# Targeting Tregs in Juvenile Idiopathic Arthritis and Juvenile Dermatomyositis—Insights From Other Diseases

**DOI:** 10.3389/fimmu.2019.00046

**Published:** 2019-01-25

**Authors:** Romy E. Hoeppli, Anne M. Pesenacker

**Affiliations:** ^1^Department of Surgery, British Columbia Children's Hospital Research Institute, University of British Columbia, Vancouver, BC, Canada; ^2^Division of Infection and Immunity, Institute of Immunity and Transplantation, University College London, London, United Kingdom

**Keywords:** regulatory T cells, juvenile idiopathic arthritis, juvenile dermatomyositis, biomarker, therapy

## Abstract

Regulatory T cells (Tregs) are believed to be dysfunctional in autoimmunity. Juvenile idiopathic arthritis (JIA) and juvenile dermatomyositis (JDM) result from a loss of normal immune regulation in specific tissues such as joints or muscle and skin, respectively. Here, we discuss recent findings in regard to Treg biology in oligo-/polyarticular JIA and JDM, as well as what we can learn about Treg-related disease mechanism, treatment and biomarkers in JIA/JDM from studies of other diseases. We explore the potential use of Treg immunoregulatory markers and gene signatures as biomarkers for disease course and/or treatment success. Further, we discuss how Tregs are affected by several treatment strategies already employed in the therapy of JIA and JDM and by alternative immunotherapies such as anti-cytokine or co-receptor targeting. Finally, we review recent successes in using Tregs as a treatment target with low-dose IL-2 or cellular immunotherapy. Thus, this mini review will highlight our current understanding and identify open questions in regard to Treg biology, and how recent findings may advance biomarkers and new therapies for JIA and JDM.

## Introduction

CD4^+^FOXP3^+^ regulatory T cells (Tregs) are a subset of CD4^+^ T helper cells present in lymphoid and non-lymphoid tissues, and are crucial for mediating tolerance to self, preventing allergies and controlling immune reactions after infections (1). They develop in the thymus or are induced in the periphery and exhibit contact-dependent and -independent mechanisms of action (1). Inactivating mutations in FOXP3 lead to multi-organ autoimmune disease [immune dysregulation, polyendocrinopathy, enteropathy, X-linked syndrome (IPEX)], highlighting the importance of Tregs (2). Importantly, it is becoming clear that the local microenvironment affects the phenotype and function of tissue-localized Tregs, which also have additional roles in repair and regeneration (3).

Treg–tissue interaction might be particular important in autoimmunity with tissue-specific presentation, such as juvenile idiopathic arthritis (JIA) and juvenile dermatomyositis (JDM). While JIA is the most common inflammatory rheumatic disease in children, JDM is rare. JIA is characterized by persistent arthritis and subtype-dependent symptoms [reviewed in (4)]. Here, we focus on polyarticular and oligoarticular JIA, which present without involvement of systemic organs or skin. JDM is characterized by inflammation of muscles and skin, resulting in muscle weakness and rashes [reviewed in (5)]. Interestingly, for both conditions researchers may take advantage of clinical sample collection from the site of inflammation: synovial fluid (SF) drained during therapeutic joint injection (JIA) and biopsies (mostly muscle, JDM). While some patients respond to therapy, others do not and studying the underlying differences may lead to better understanding and treatments.

Here, we discuss recent advances in the understanding of Treg biology in oligo-/ polyarticular JIA and JDM, and what we can learn about Treg-related disease mechanisms, treatments and biomarkers from other diseases.

## Altered Tregs in JIA and JDM

The phenotype of CD4^+^FOXP3^+^ Tregs in JIA has been considerably characterized in the past (6) with the molecular roles of FOXP3 in JIA reviewed by Copland and Bending in this special collection (7). It is now clear that the Treg TCR (T cell receptor) repertoire is highly restricted in JIA, both at the site of inflammation (8–11) and in circulation (10, 12). Interestingly, in blood only Tregs but not conventional CD4^+^ non-Treg cells (Tconv) are more clonal (10, 12). Some suggest that the TCR repertoires of Tregs from SF and peripheral blood (PB) significantly overlap (8), while others only found a very small overlap (9, 11). These differences might be explained by different sequencing depth and analysis strategies and/or by different Treg subsets studied: total (11) or effector Tregs defined by HLA-DR (8) or CD161 expression (9). Further, one study found that SF Tregs, but not Tconv, share specificity at an amino acid sequence level among different patients (10), suggesting disease-associated Treg clones might foster JIA.

Besides a restricted TCR repertoire, Tregs from the JIA inflammatory sites show unstable FOXP3 and CD25 (13), altered homing markers (9), cytokine production (6, 9), deficiency in specific chemokine production (14), and low responsiveness to IL-2 (13)—indicating impaired Treg function in JIA. Nevertheless, many reports found that JIA SF and PB Tregs are fully demethylated (8, 13), thus committed to the Treg-lineage, and suppressive *in vitro* (6, 8, 9, 13, 15). Hence, JIA Tregs are likely functioning inappropriately or insufficiently in the context of the inflammatory microenvironment. Interestingly, adding SF to *in vitro* cultures can both increase/stabilize Treg FOXP3 expression (11, 16) and *in situ* induce effector T cells to be resistant to Treg-mediated suppression *ex vivo* (17, 18). Thus, more research is needed to decipher the effects of the inflammatory microenvironment on Treg function.

In comparison, we know little about the contribution of Tregs to JDM pathogenesis. Similar to JIA, the Treg repertoire is restricted with a lack of diversity (12). FOXP3^+^ Tregs were found to be enriched in JDM muscle compared to muscle tissue from patients with Duchenne muscular dystrophy (19). Since the latter is already enriched in Tregs compared to normal muscle (20), this suggests a hyper-enrichment in JDM in response to autoimmune inflammation. PB Tregs of active JDM also appear less suppressive *in vitro* with decreased expression of CTLA4 (19). Adult DM/ polymyositis muscle biopsies are also enriched with Tregs (21). Interestingly, both Treg and effector T cell numbers decreased post immunosuppressive therapy in adult myositis, suggesting that Treg enrichment is a response to inflammation. However, juvenile and adult DM have different clinical presentation (22) and JDM PB express more Th17-type and FOXP3 transcripts (23). JDM and other myopathies are characterized by a type 1 IFN signature (24–26) and interferons may be a potential therapeutic target (27), but their effects on Tregs remain to be investigated.

Tregs are crucial in resolving muscle injury in animal studies (28) and Treg-deficient mice develop more severe myopathies in response to antigen, while adoptive Treg transfer prevents inflammation (29, 30). Thorough immune-profiling recently revealed pan-tissue and tissue-specific signatures and enhancers of murine Tregs (31). The muscle Treg signature was highly enriched in cell cycle genes, showed a dynamic response to injury and was more similar to circulating Treg signatures than to other tissue Tregs (31), indicating that muscle Tregs might acutely infiltrate muscle and are not necessarily long-term resident cells. While myopathy is a defining characteristic of JDM, skin inflammation and rash are other symptoms (5). Skin-resident Tregs are crucial for immune homeostasis (3) and have been characterized in health and various disease settings (32). However, studies on JDM-affected skin are lacking, and more work is needed to characterize JDM skin-resident Tregs.

## Tregs as a Biomarker?

**Figure 1:**
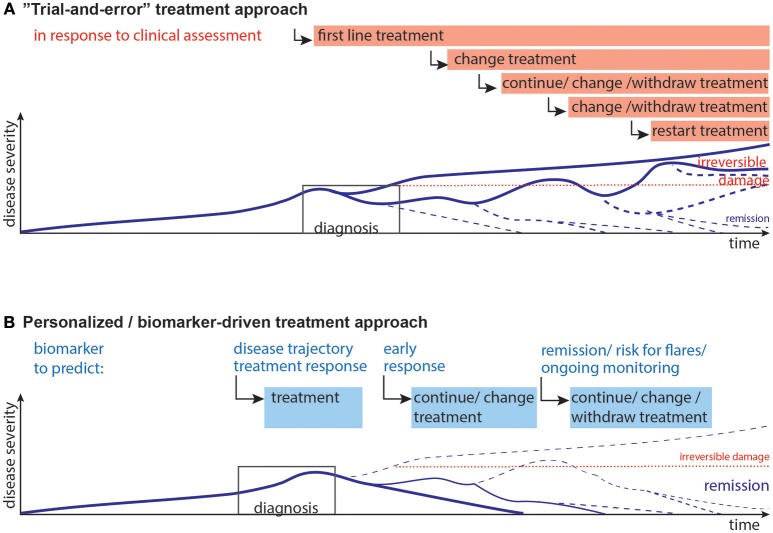
Current and desired disease progression models. **(A)** Current “Trial-and-error” model: Upon diagnosis the first line of treatment is started, which may lead to remission, or partial remission. Often, a second, third or fourth treatment strategy needs to be implemented when the previous treatments are not effective. Choice of treatments is guided by previous experience, e.g., upon presentation non-steroidal anti-inflammatory drugs with glucocorticoids are used, after a couple of month many patients are switched to methotrexate as first disease modifying therapy, often followed by anti-TNF-α agents with/without methotrexate (4). This disease progression and subsequent staggering of therapy can result in irreversible damage and long-term therapy. **(B)** Desired personalized/biomarker-driven model: Biomarkers could be used for prediction and aid decisions at the following stages: disease trajectory, selection of treatment, early response to therapy, remission/minimal disease activity and the risk of flare upon withdrawal of therapy, ongoing monitoring of immune activity and risk of flares. Biomarkers might thus contribute to more efficient therapy, pre-empt flares, and minimize short- and long-term effects of flares and reduce long-term damage. Lines represent models of disease progression, with line thickness representing frequency estimates.

JIA and JDM can exhibit an unpredictable disease course. While mounting evidence indicates that an early aggressive treatment is best for severe disease (4, 27, 33, 34), the disease course is unpredictable at presentation. Additionally, due to potential short- and long-term side effects children should not be exposed to unnecessary medication. Unfortunately, once a patient appears to be in clinical remission (on or off medications), disease may flare without any notice or obvious trigger (Figure 1A). Indeed, among JIA patients who are in clinical remission, 30–50% experience flares (35, 36).

Hence, reliable biomarkers need to predict (i) the future disease course, (ii) treatment response, and (iii) the safety for medication withdrawal during clinical remission (Figure 1B).

Inflammation markers in the serum can indicate disease activity and potentially treatment response in JIA [reviewed in (36)]. In JDM, histology of biopsies and myositis-specific auto-antibodies can indicate future disease severity or complications [reviewed in (27)].

Only a few putative biomarkers probe the immunoregulatory balance in autoimmune arthritis and myopathies. The frequency of inflammation-associated Tregs (HLA-DR^+^) in PB was proposed as a biomarker for disease activity in arthritis (8). TCR sequence overlap of these PB HLA-DR^+^ Tregs with SF Tregs in JIA, and an increase of HLA-DR^+^ Tregs in active rheumatoid arthritis (RA) were found. Low expression of the immunoregulatory receptor CD39 has been suggested as an indicator of methotrexate resistance in RA (37). Also, response to the TNF-α blocker adalimumab could be predicted by a Treg increase in PBMCs from RA patients cultured with adalimumab prior to treatment (38). Finally, the soluble form of the high affinity IL-2 receptor α chain (CD25), crucial for Treg phenotype and function, might be a biomarker for adult myositis disease activity (39).

In the recent past, gene signatures have been defined as multi-parameter biomarkers. Thus, far, efforts to define JIA immune-based gene biomarkers have focused on whole genome expression profiling (40–42) and epigenomic signatures (43, 44). JIA displays an altered immune signature, which changes during remission, but does not return to a state comparable to healthy controls (41, 42). Myositis is characterized by type 1 IFN signatures (27). While interesting and highlighting potential disease mechanisms, whole-genome/exome expression profiling is not feasible for routine clinical practice due to cost, logistics and data interpretation. We have recently developed a Treg gene signature associated with Treg competency using the clinically-applicable multiplex platform nanoString (45). NanoString is fast and fewer than 10,000 lysed cells are sufficient without the need to purify RNA. Although the proportion of Tregs that express FOXP3 was similar between type 1 diabetes (T1D) and controls, there was a significant change in their Treg signature (45). Future work will elucidate whether the Treg gene signature may also be used as a biomarker in JIA and other autoimmune conditions.

In summary, some progress has been made, but more biomarkers are needed for biological disease activity, prognosis, treatment success, and risk of flares. Further, a consensus of criteria to describe active/inactive disease is needed to better estimate the currently widely variable incidence of clinically inactive JIA disease (46). For JDM, a comprehensive set of criteria to assess disease activity and damage has been proposed (47).

## Tregs as Therapeutic Target/Tool?

Convincing evidence demonstrates that functioning Tregs are crucial to prevent autoimmunity and our understanding of how different immunotherapies affect Tregs has improved.

### (Unforeseen) Treg Effects of Immuno-Therapy

High levels of TNF-α in the inflamed JIA joint (32, 48) offer a clear rationale for anti-TNF therapy in JIA with marked success (4, 49). Anti-TNF therapy has also been used in refractory JDM (50), but with mixed evidence for its effectiveness (27, 51–53). Blocking TNF-α can, however, also elicit further autoimmune responses, particular in the skin and muscle (54–57). TNF-α itself can have both positive and negative effects on Tregs (32, 58, 59). Interestingly, the negative effects are found especially in inflamed joints (32, 58, 60), whereas positive effects of TNF-α on Treg function were reported using healthy human cells or in mice (58, 61–64). TNF-α has two receptors CD120a (TNFR1) and CD120b (TNFR2) (58). CD120b may mediate the pro-Treg functions of TNF-α, including Treg proliferation, stabilizing Tregs, and preventing disease in mouse models (58, 62–64). Little is known about the effects of ligation of CD120a in Tregs, but some research suggests targeting CD120a while sparing CD120b-TNF-interaction can alleviate collagen-induced arthritis (65). In RA, adalimumab has been shown to enhance Treg frequency and potency via CD120b (38, 66, 67). Etanercept, a soluble CD120b as TNF-α blocker, instead might predominantly affect effector T cells, by reversing their resistance to suppression in JIA (68). Unfortunately, a considerable group of JIA/JDM patients do not respond to anti-TNF therapy (27, 49, 53) and anti-TNF agents are immunogenic (69), with 50% of patients developing anti-drug antibodies leading to resistance to therapy and disease progression.

Ustekinumab is another potentially attractive anti-cytokine therapy which targets the p40 subunit of IL-12 and IL-23, key cytokines driving Th1, Th17, and Th17.1, (ex-)Th17 cells with a Th1-like phenotype, function (70–74). Ustekinumab is well-tolerated in adult and pediatric patients for treating psoriasis, psoriatic arthritis, systemic lupus erythematosus (SLE), and Crohn's disease (70, 71, 75–77), and has shown lower immunogenicity compared to most anti-TNF agents (69). Th17.1 are enriched in JIA (72, 73), and ustekinumab therapy had some success in enthesitis-related JIA (78), psoriatic arthritis (69, 79) and is in trial for various rheumatological diseases (80). While no imbalance in IL-17 has been established in JDM (19), Th17.1 have not been investigated. Ustekinumab has been suggested as a potential therapy for JDM, and a case of JDM with psoriasis was treated successfully with ustekinumab (81). Due to the reciprocal relationship between Th17 and Tregs (82), Tregs might also be affected by ustekinumab therapy, and this was indeed suggested in a case report of giant cell arteritis (83) and in T1D treated with ustekinumab (NCT02117765; Pesenacker et al.).

IL-6 also drives inflammatory environments, including skewing the Treg/Th17 balance toward Th17 (71). Anti-IL-6 receptor therapy (tocilizumab) increases Treg frequency and numbers in RA (71). IL-6 has also been implicated in JIA and JDM (11, 17, 84, 85) and is used in polyarticular, extended oligoarticular, systemic JIA (49), and refractory JDM (50), but mechanistic studies in pediatric disease are lacking.

Whether drugs such as ustekinumab and tocilizumab act on Tregs directly or through changing the microenvironment is unclear. Human Tregs can express the receptors for IL-6 (86), IL-12 (87), and IL-23 (88), but evidence for direct drug action on Tregs is lacking.

Alternatively, co-receptors can be targeted to manipulate the immunoregulatory balance. Initially established for cancer therapy (checkpoint blockade), mimicking checkpoints such as CTLA4 (CTLA4-Ig, abatacept, belatacept) is used as treatment for autoimmunity. Abatacept has been shown to be safe and effective in oligo- and polyarticular JIA (49, 89–92), adult DM/polymyositis (34, 53), a case report of steroid-sparing abatacept in complex JDM (93) and a trial in JDM is underway (27). A reduction of the T cell activation state is the main reported effect of abatacept (90, 94–96). Surprisingly, the majority of studies found abatacept decreases the frequency of Tregs (90, 95, 97–99), with some studies showing an increase in function (99). On the other hand, increased Treg frequency, but decreased activity after abatacept therapy in RA was demonstrated (100). In muscle tissue of adult DM/polymyositis, more Tregs were found following abatacept (34), suggesting that abatacept treatment could change Treg localization. Other co-receptor targeting therapies are in use/development for malignancies (e.g., anti-PD1, anti-TIM3, anti-TIGIT, etc.) and these pathways might be useful targets in autoimmunity.

### Treg (-Targeted) Therapy

Adoptive transfer of Tregs has been shown to be safe and possibly effective at reducing inflammation, inducing transplant tolerance, preventing graft-vs.-host disease (GVHD) and treating autoimmunity [reviewed in (101)].

Important considerations for Treg-therapy currently under investigation are the source of therapeutic cells, antigen-specificity and possibly tailoring homing characteristics for improved activity. Isolating and expanding sufficient numbers of Tregs from patients awaiting transplantation, under immunosuppression or with autoimmune disease is feasible and can restore their function (101–103), although achieving clinically relevant Treg numbers from pediatric JIA and JDM patients might prove challenging. Third-party Tregs from umbilical cord blood have been found safe and possibly effective as GVHD prophylaxis in adults (104, 105) and pediatric thymus—routinely removed during pediatric cardiac surgery—might be a plentiful source for highly functional therapeutic Tregs (106, 107). Antigen-specific Tregs are more effective than polyclonal Tregs for therapy and with recent successes of chimeric antigen receptor (CAR) T effector therapies for cancer, there has been a surge to adapt this technology to generate CAR-Tregs [reviewed in (108)]. While generation of antigen-specific Tregs recognizing allogeneic HLA-molecules is relatively straightforward in transplantation, generation of CAR-Tregs for autoimmunity without known antigen (i.e., JIA) might be difficult. Still, CAR-Tregs reacting with antigen found at the site of inflammation (i.e., JIA joints or JDM muscle) could activate Tregs locally. Alternatively, Tregs could be conditioned *in vitro* to home to specific sites (107) or Tregs could be injected locally, as shown with intra-dermal injection of Tregs to inhibit murine allograft skin inflammation (109).

Since Treg cell therapies are challenging and expensive, targeting Treg expansion *in vivo* might be more feasible for conditions such as JIA and JDM. The most promising advances of non-cellular therapies targeting Tregs have been low-dose IL-2, IL-2 complexes, or IL-2 bio-similars (110–112). While high doses of IL-2 stimulate mainly effector cells, low-dose IL-2 [0.3–3 × 10^6^ units/day (112)] skews the response toward Tregs. Low-dose IL-2 increases the frequency of activated, functional and fully demethylated CD25^+^FOXP3^+^ Tregs (113–115) and induces STAT5 phosphorylation *in vivo* (114, 115). Low-dose IL-2 therapy has been deemed safe and successful in the treatment of T1D (112, 114, 115), GVHD (116, 117), and SLE (113). Indeed, low-dose IL-2 therapy rescued Tregs with low levels of CD25 in SLE (113), indicating that it might also rescue JIA Tregs with low CD25 expression (13). To further fine-tune specificity or increase the half-life of IL-2, IL-2 complexes, and bio-similars are in development (110, 111, 118); these expand Tregs and induce phosphorylated STAT5 *in vitro, in vivo*, and prevent disease in animal models (118–121), including resolution of muscular dystrophy (20). Covalently linking IL-2 to anti-IL-2 (122), to non-FcRγ-binding human IgG1 (123) or CD25 (124) may enhance potential clinical application by mitigating the risk of *in vivo* dissociation of complexes.

However, increasing Treg numbers alone might not be sufficient to overcome the highly inflammatory environment and effector cell resistance. Thus, to achieve sustained remission combination-therapy might be necessary to reduce the inflammatory milieu paralleled with boosting Tregs to maintain a renewed tolerance.

## Concluding Remarks

**Figure 2:**
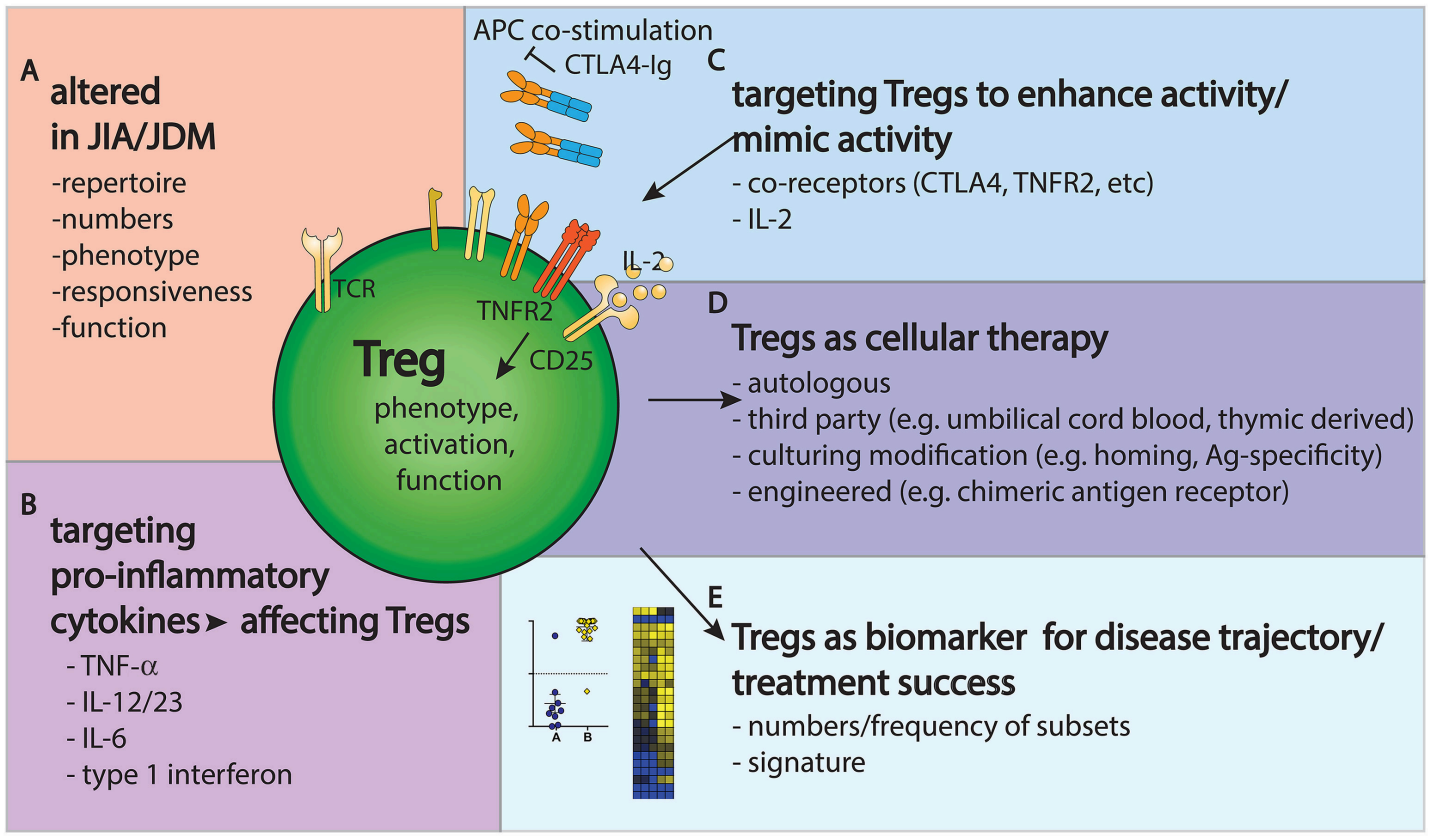
Tregs provide challenges and opportunities in JIA/JDM pathogenesis, treatment and monitoring. **(A)** Tregs are altered in repertoire, phenotype and frequency in JIA/JDM, particularly at the site of inflammation. **(B–D)** Treatment options to restore the immunoregulatory balance include targeting pro-inflammatory cytokines **(B)**, targeting Tregs to enhance their activity **(C)** or using Tregs as a cellular therapy **(D)**. **(E)** Further, changes in Treg gene signatures could aid as biomarkers to measure or predict disease/treatment response.

Taken together, it is clear that Tregs present challenges and opportunities in JIA and JDM research and clinical management (Figure 2). Their phenotype and function are clearly altered in JIA and JDM, targeting them might improve disease outcome and Tregs could be used as biomarkers to gage the state and progress of disease.

The role of the microenvironment on Treg function and phenotype in JIA- and JDM-affected tissues remains to be explored further. Researchers should take advantage of biopsies taken for clinical diagnosis (JDM) and SF aspirated during therapeutic joint injections (JIA). Novel techniques, such as single cell sequencing, multidimensional mass/flow cytometry and microscopy, will aid using clinical samples to their full potential (125–127). Additionally, co-culture with SF or muscle-derived cells could highlight how the microenvironment affects Tregs. Since JDM in particular is a rare disease, collaborations between groups are crucial to increase sample size for fundamental research, biomarker-finding and -validation studies and controlled treatment trials. This could be achieved by consortiums similar to juvenile diabetes research foundation (JDRF) biomarker working group for T1D (114) and the immune tolerance network trials (128).

While there is progress toward unified measures of disease activity (46, 47), these will need to be tested and verified, followed by development of feasible, reliable and cost-effective biomarkers to predict disease activity, risk of flare and ideal treatment strategies. The ultimate goal, aided by biomarkers, is to go from a trial-and-error treatment approach toward a more efficient and personalized medicine approach with more patients achieving drug-free remission without major long-term disabilities (Figure 1).

Success of various agents affecting the immunoregulatory balance in other diseases point to potential uses in JIA and JDM. Any (new) therapy will need to be considered in regards to both effector cells AND Tregs, since some therapies might have unexpected effects on Tregs. Thus, it is important to continuously build our understanding of how various agents affect the immunoregulatory balance.

In conclusion, important recent advances might lead to valid future contributions to the widened arsenal of treatment options available to restore the immunoregulatory balance in a heterogeneous disease spectrum.

## Author Contributions

AP conceived the review. RH and AP reviewed the literature and co-wrote the manuscript.

### Conflict of Interest Statement

The authors declare that the research was conducted in the absence of any commercial or financial relationships that could be construed as a potential conflict of interest.

## References

[1] Sakaguchi S Miyara M Costantino CM Hafler DA. FOXP3+ regulatory T cells in the human immune system. Nat Rev Immunol. (2010) 10:490–500. 10.1038/nri2785 20559327

[2] Bacchetta R Barzaghi F Roncarolo MG. From IPEX syndrome to FOXP3 mutation: a lesson on immune dysregulation. Ann N Y Acad Sci. (2016) 1417:5–22. 10.1111/nyas.13011 26918796

[3] Li J Tan J Martino MM Lui KO. Regulatory T-Cells: potential regulator of tissue repair and regeneration. Front Immunol. (2018) 9:585. 10.3389/fimmu.2018.00585 29662491 PMC5890151

[4] Crayne CB Beukelman T. Juvenile idiopathic arthritis: oligoarthritis and polyarthritis. Pediatr Clin North Am. (2018) 65:657–74. 10.1016/j.pcl.2018.03.005 30031492

[5] Rider LG Nistala K. The juvenile idiopathic inflammatory myopathies: pathogenesis, clinical and autoantibody phenotypes, and outcomes. J Intern Med. (2016) 280:24–38. 10.1111/joim.12444 27028907 PMC4914449

[6] Pesenacker AM Wedderburn LR. T regulatory cells in childhood arthritis–novel insights. Expert Rev Mol Med. (2013) 15:e13. 10.1017/erm.2013.14 24294966

[7] Copland A Bending D. Foxp3 molecular dynamics in treg in juvenile idiopathic arthritis. Front Immunol. (2018) 9:2273. 10.3389/fimmu.2018.02273 30333832 PMC6175987

[8] Rossetti M Spreafico R Consolaro A Leong JY Chua C Massa M. TCR repertoire sequencing identifies synovial Treg cell clonotypes in the bloodstream during active inflammation in human arthritis. Ann Rheum Dis. (2017) 76:435–41. 10.1136/annrheumdis-2015-208992 27311837 PMC5284348

[9] Duurland CL Brown CC O'Shaughnessy RF Wedderburn LR. CD161(+) Tconv and CD161(+) Treg share a transcriptional and functional phenotype despite limited overlap in TCRbeta repertoire. Front Immunol. (2017) 8:103. 10.3389/fimmu.2017.00103 28321213 PMC5337494

[10] Henderson LA Volpi S Frugoni F Janssen E Kim S Sundel RP. Next-generation sequencing reveals restriction and clonotypic expansion of treg cells in juvenile idiopathic arthritis. Arthritis Rheumatol. (2016) 68:1758–68. 10.1002/art.39606 26815131 PMC5398095

[11] Bending D Giannakopoulou E Lom H Wedderburn LR. Synovial regulatory T cells occupy a discrete TCR niche in human arthritis and require local signals to stabilize FOXP3 protein expression. J Immunol. (2015) 195:5616–24. 10.4049/jimmunol.1500391 26561546 PMC4671090

[12] Delemarre EM van den Broek T Mijnheer G Meerding J Wehrens EJ Olek S. Autologous stem cell transplantation aids autoimmune patients by functional renewal and TCR diversification of regulatory T cells. Blood (2016) 127:91–101. 10.1182/blood-2015-06-649145 26480932

[13] Bending D Pesenacker AM Ursu S Wu Q Lom H Thirugnanabalan B. Hypomethylation at the regulatory T cell-specific demethylated region in CD25hi T cells is decoupled from FOXP3 expression at the inflamed site in childhood arthritis. J Immunol. (2014) 193:2699–708. 10.4049/jimmunol.1400599 25092890 PMC4157061

[14] Patterson SJ Pesenacker AM Wang AY Gillies J Mojibian M Morishita K. T regulatory cell chemokine production mediates pathogenic T cell attraction and suppression. J Clin Invest. (2016) 126:1039–51. 10.1172/JCI83987 26854929 PMC4767359

[15] Bulatovic Calasan M Vastert SJ Scholman RC Verweij F Klein M Wulffraat NM Methotrexate treatment affects effector but not regulatory T cells in juvenile idiopathic arthritis. Rheumatology (2015) 54:1724–34. 10.1093/rheumatology/kev101 25877908

[16] Ohl K Nickel H Moncrieffe H Klemm P Scheufen A Foll D. The transcription factor CREM drives an inflammatory phenotype of T cells in oligoarticular juvenile idiopathic arthritis. Pediatr Rheumatol Online J. (2018) 16:39. 10.1186/s12969-018-0253-x 29925386 PMC6011589

[17] Wehrens EJ Mijnheer G Duurland CL Klein M Meerding J van Loosdregt J. Functional human regulatory T cells fail to control autoimmune inflammation due to PKB/c-akt hyperactivation in effector cells. Blood (2011) 118:3538–48. 10.1182/blood-2010-12-328187 21828127

[18] Haufe S Haug M Schepp C Kuemmerle-Deschner J Hansmann S Rieber N. Impaired suppression of synovial fluid CD4+CD25- T cells from patients with juvenile idiopathic arthritis by CD4+CD25+ Treg cells. Arthritis Rheum. (2011) 63:3153–62. 10.1002/art.30503 21702013

[19] Vercoulen Y Bellutti Enders F Meerding J Plantinga M Elst EF Varsani H. Increased presence of FOXP3+ regulatory T cells in inflamed muscle of patients with active juvenile dermatomyositis compared to peripheral blood. PLoS ONE (2014) 9:e105353. 10.1371/journal.pone.0105353 25157414 PMC4144849

[20] Villalta SA Rosenthal W Martinez L Kaur A Sparwasser T Tidball JG. Regulatory T cells suppress muscle inflammation and injury in muscular dystrophy. Sci Transl Med. (2014) 6:258ra142. 10.1126/scitranslmed.3009925 25320234 PMC4889432

[21] Pandya JM Loell I Hossain MS Zong M Alexanderson H Raghavan S. Effects of conventional immunosuppressive treatment on CD244+ (CD28null) and FOXP3+ T cells in the inflamed muscle of patients with polymyositis and dermatomyositis. Arthritis Res Ther. (2016) 18:80. 10.1186/s13075-016-0974-5 27039301 PMC4818535

[22] Tansley SL McHugh NJ Wedderburn LR. Adult and juvenile dermatomyositis: are the distinct clinical features explained by our current understanding of serological subgroups and pathogenic mechanisms? Arthritis Res Ther. (2013) 15:211. 10.1186/ar4198 23566358 PMC3672700

[23] Lopez De Padilla CM Crowson CS Hein MS Pendegraft RS Strausbauch MA Niewold TB. Gene expression profiling in blood and affected muscle tissues reveals differential activation pathways in patients with new-onset juvenile and adult dermatomyositis. J Rheumatol. (2017) 44:117–24. 10.3899/jrheum.160293 27803134 PMC5268083

[24] Gitiaux C Latroche C Weiss-Gayet M Rodero MP Duffy D Bader-Meunier B. Myogenic progenitor cells exhibit type I interferon-driven proangiogenic properties and molecular signature during juvenile dermatomyositis. Arthritis Rheumatol. (2018) 70:134–45. 10.1002/art.40328 28941175

[25] Greenberg SA. A gene expression approach to study perturbed pathways in myositis. Curr Opin Rheumatol. (2007) 19:536–41. 10.1097/BOR.0b013e3282efe261 17917532

[26] Zhu W Streicher K Shen N Higgs BW Morehouse C Greenlees L. Genomic signatures characterize leukocyte infiltration in myositis muscles. BMC Med Genomics (2012) 5:53. 10.1186/1755-8794-5-53 23171592 PMC3541209

[27] Wu Q Wedderburn LR McCann LJ. Juvenile dermatomyositis: latest advances. Best Pract Res Clin Rheumatol. (2017) 31:535–57. 10.1016/j.berh.2017.12.003 29773272

[28] Deyhle MR Hyldahl RD. The role of T lymphocytes in skeletal muscle repair from traumatic and contraction-induced injury. Front Physiol. (2018) 9:768. 10.3389/fphys.2018.00768 29973887 PMC6019499

[29] Young NA Sharma R Friedman AK Kaffenberger BH Bolon B Jarjour WN. Aberrant muscle antigen exposure in mice is sufficient to cause myositis in a Treg cell-deficient milieu. Arthritis Rheum. (2013) 65:3259–70. 10.1002/art.38184 24022275 PMC4033530

[30] Allenbach Y Solly S Gregoire S Dubourg O Salomon B Butler-Browne G. Role of regulatory T cells in a new mouse model of experimental autoimmune myositis. Am J Pathol. (2009) 174:989–98. 10.2353/ajpath.2009.080422 19218348 PMC2665758

[31] DiSpirito JR Zemmour D Ramanan D Cho J Zilionis R Klein AM. Molecular diversification of regulatory T cells in nonlymphoid tissues. Sci Immunol. (2018) 3:eaat5861. 10.1126/sciimmunol.aat5861 30217811 PMC6219455

[32] Pesenacker AM Broady R Levings MK. Control of tissue-localized immune responses by human regulatory T cells. Eur J Immunol. (2015) 45:333–43. 10.1002/eji.201344205 25378065

[33] Wallace CA Ringold S Bohnsack J Spalding SJ Brunner HI Milojevic D. Extension study of participants from the trial of early aggressive therapy in juvenile idiopathic arthritis. J Rheumatol. (2014) 41:2459–65. 10.3899/jrheum.140347 25179849

[34] Tjarnlund A Tang Q Wick C Dastmalchi M Mann H Tomasova Studynkova J. Abatacept in the treatment of adult dermatomyositis and polymyositis: a randomised, phase IIb treatment delayed-start trial. Ann Rheum Dis. (2018) 77:55–62. 10.1136/annrheumdis-2017-211751 28993346

[35] Wallace CA Huang B Bandeira M Ravelli A Giannini EH. Patterns of clinical remission in select categories of juvenile idiopathic arthritis. Arthritis Rheum. (2005) 52:3554–62. 10.1002/art.21389 16255044

[36] Duurland CL Wedderburn LR. Current developments in the use of biomarkers for juvenile idiopathic arthritis. Curr Rheumatol Rep. (2014) 16:406. 10.1007/s11926-013-0406-3 24445961 PMC3930839

[37] Peres RS Liew FY Talbot J Carregaro V Oliveira RD Almeida SL. Low expression of CD39 on regulatory T cells as a biomarker for resistance to methotrexate therapy in rheumatoid arthritis. Proc Natl Acad Sci USA. (2015) 112:2509–14. 10.1073/pnas.1424792112 25675517 PMC4345589

[38] Nguyen DX Cotton A Attipoe L Ciurtin C Doré CJ Ehrenstein MR. Regulatory T cells as a biomarker for response to adalimumab in rheumatoid arthritis. J Allergy Clin Immunol. (2018) 142:978–80.e979. 10.1016/j.jaci.2018.04.026 29935955 PMC6127034

[39] Tournadre A Dubost JJ Soubrier M Ruivard M Souteyrand P Schmidt J. Soluble IL-2 receptor: a biomarker for assessing myositis activity. Dis Markers (2014) 2014:472624. 10.1155/2014/472624 24648607 PMC3932274

[40] Cui A Quon G Rosenberg AM Yeung RS Morris Q Consortium BS. Gene expression deconvolution for uncovering molecular signatures in response to therapy in juvenile idiopathic arthritis. PLoS ONE (2016) 11:e0156055. 10.1371/journal.pone.0156055 27244050 PMC4887077

[41] Jiang K Frank M Chen Y Osban J Jarvis JN. Genomic characterization of remission in juvenile idiopathic arthritis. Arthritis Res Ther. (2013) 15:R100. 10.1186/ar4280 24000795 PMC4062846

[42] Moncrieffe H Bennett MF Tsoras M Luyrink LK Johnson AL Xu H. Transcriptional profiles of JIA patient blood with subsequent poor response to methotrexate. Rheumatology (2017) 56:1542–51. 10.1093/rheumatology/kex206 28582527 PMC5850489

[43] Peeters JG Vervoort SJ Tan SC Mijnheer G de Roock S Vastert SJ. Inhibition of super-enhancer activity in autoinflammatory site-derived T Cells reduces disease-associated gene expression. Cell Rep. (2015) 12:1986–96. 10.1016/j.celrep.2015.08.046 26387944

[44] Spreafico R Rossetti M Whitaker JW Wang W Lovell DJ Albani S. Epipolymorphisms associated with the clinical outcome of autoimmune arthritis affect CD4+ T cell activation pathways. Proc Natl Acad Sci USA. (2016) 113:13845–50. 10.1073/pnas.1524056113 27849614 PMC5137722

[45] Pesenacker AM Wang AY Singh A Gillies J Kim YW Piccirillo CA A Treg gene signature is a specific and sensitive biomarker to identify children with new onset type 1 diabetes. Diabetes (2016) 65:1031–9. 10.2337/db15-0572 26786322

[46] Shoop-Worrall SJW Verstappen SMM Baildam E Chieng A Davidson J Foster H. How common is clinically inactive disease in a prospective cohort of patients with juvenile idiopathic arthritis? The importance of definition. Ann Rheum Dis. (2017) 76:1381–8. 10.1136/annrheumdis-2016-210511 28389553 PMC5738598

[47] McCann LJ Pilkington CA Huber AM Ravelli A Appelbe D Kirkham JJ. Development of a consensus core dataset in juvenile dermatomyositis for clinical use to inform research. Ann Rheum Dis. (2018) 77:241–50. 10.1136/annrheumdis-2017-212141 29084729 PMC5816738

[48] de Jager W Hoppenreijs EP Wulffraat NM Wedderburn LR Kuis W Prakken BJ. Blood and synovial fluid cytokine signatures in patients with juvenile idiopathic arthritis: a cross-sectional study. Ann Rheum Dis. (2007) 66:589–98. 10.1136/ard.2006.061853 17170049 PMC1954617

[49] Shepherd J Cooper K Harris P Picot J Rose M. The clinical effectiveness and cost-effectiveness of abatacept, adalimumab, etanercept and tocilizumab for treating juvenile idiopathic arthritis: a systematic review and economic evaluation. Health Technol Assess. (2016) 20:1–222. 10.3310/hta20340 27135404 PMC4867422

[50] Spencer CH Rouster-Stevens K Gewanter H Syverson G Modica R Schmidt K. Biologic therapies for refractory juvenile dermatomyositis: five years of experience of the childhood arthritis and rheumatology research alliance in North America. Pediatr Rheumatol Online J. (2017) 15:50. 10.1186/s12969-017-0174-0 28610606 PMC5470177

[51] Rouster-Stevens KA Ferguson L Morgan G Huang CC Pachman LM. Pilot study of etanercept in patients with refractory juvenile dermatomyositis. Arthritis Care Res (2014) 66:783–7. 10.1002/acr.22198 24127327

[52] Wang CR. Successful treatment of refractory juvenile dermatomyositis with adalimumab. J Clin Rheumatol. (2017) 23:174–5. 10.1097/RHU.0000000000000514 28333871

[53] Sasaki H Kohsaka H. Current diagnosis and treatment of polymyositis and dermatomyositis. Mod Rheumatol. 28:913–21. 10.1080/14397595.2018.1467257 29669460

[54] Omran NE Noorwali AA. Nephritis, cerebritis, and myositis after adalimumab therapy in a patient with rheumatoid arthritis: a case report. Int J Gen Med. (2018) 11:151–4. 10.2147/IJGM.S154835 29692623 PMC5903491

[55] Brunasso AM Aberer W Massone C. New onset of dermatomyositis/polymyositis during anti-TNF-alpha therapies: a systematic literature review. ScientificWorldJournal (2014) 2014:179180. 10.1155/2014/179180 24600322 PMC3926249

[56] Liu SW Velez NF Lam C Femia A Granter SR Townsend HB. Dermatomyositis induced by anti-tumor necrosis factor in a patient with juvenile idiopathic arthritis. JAMA Dermatol. (2013) 149:1204–8. 10.1001/jamadermatol.2013.5220 23986394

[57] Ko JM Gottlieb AB Kerbleski JF. Induction and exacerbation of psoriasis with TNF-blockade therapy: a review and analysis of 127 cases. J Dermatolog Treat. (2009) 20:100–8. 10.1080/09546630802441234 18923992

[58] Goldstein JD Perol L Zaragoza B Baeyens A Marodon G Piaggio E. Role of cytokines in thymus- versus peripherally derived-regulatory T cell differentiation and function. Front Immunol. (2013) 4:155. 10.3389/fimmu.2013.00155 23801992 PMC3685818

[59] Nie H Zheng Y Li R Zhang J. Reply to suppressive activity of human regulatory T cells is maintained in the presence of TNF. Nat Med. (2016) 22:18–9. 10.1038/nm.4018 26735403

[60] Wang J van Dongen H Scherer HU Huizinga TW Toes RE. Suppressor activity among CD4+,CD25++ T cells is discriminated by membrane-bound tumor necrosis factor alpha. Arthritis Rheum. (2008) 58:1609–18. 10.1002/art.23460 18512781

[61] Zaragoza B Chen X Oppenheim JJ Baeyens A Gregoire S Chader D. Suppressive activity of human regulatory T cells is maintained in the presence of TNF. Nat Med. (2016) 22:16–7. 10.1038/nm.4019 26735402 PMC6345394

[62] Wang J Ferreira R Lu W Farrow S Downes K Jermutus L. TNFR2 ligation in human T regulatory cells enhances IL2-induced cell proliferation through the non-canonical NF-kappaB pathway. Sci Rep. (2018) 8:12079. 10.1038/s41598-018-30621-4 30104686 PMC6089958

[63] Fischer R Proske M Duffey M Stangl H Martinez GF Peters N. Selective activation of tumor necrosis factor receptor ii induces antiinflammatory responses and alleviates experimental arthritis. Arthritis Rheumatol. (2018) 70:722–35. 10.1002/art.40413 29342501

[64] Lamontain V Schmid T Weber-Steffens D Zeller D Jenei-Lanzl Z Wajant H Stimulation of TNF receptor type 2 expands regulatory T cells and ameliorates established collagen-induced arthritis in mice. Cell Mol Immunol. (2018) 16:76–85. 10.1038/cmi.2017.138 PMC6318277 29375132

[65] McCann FE Perocheau DP Ruspi G Blazek K Davies ML Feldmann M. Selective tumor necrosis factor receptor I blockade is antiinflammatory and reveals immunoregulatory role of tumor necrosis factor receptor II in collagen-induced arthritis. Arthritis Rheumatol. (2014) 66:2728–38. 10.1002/art.38755 24965881

[66] McGovern JL Nguyen DX Notley CA Mauri C Isenberg DA Ehrenstein MR. Th17 cells are restrained by Treg cells via the inhibition of interleukin-6 in patients with rheumatoid arthritis responding to anti-tumor necrosis factor antibody therapy. Arthritis Rheum. (2012) 64:3129–38. 10.1002/art.34565 22674488

[67] Nguyen DX Ehrenstein MR Anti-TNF drives regulatory T cell expansion by paradoxically promoting membrane TNF-TNF-RII bindin–g in rheumatoid arthritis. J Exp Med. (2016) 213:1241–53. 10.1084/jem.20151255 27270893 PMC4925013

[68] Petrelli A Wehrens EJ Scholman RC Prakken BJ Vastert SJ van Wijk F. Self-sustained resistance to suppression of CD8+ Teff cells at the site of autoimmune inflammation can be reversed by tumor necrosis factor and interferon-gamma blockade. Arthritis Rheumatol. (2016) 68:229–36. 10.1002/art.39418 26360332

[69] Strand V Balsa A Al-Saleh J Barile-Fabris L Horiuchi T Takeuchi T. Immunogenicity of biologics in chronic inflammatory diseases: a systematic review. BioDrugs (2017) 31:299–316. 10.1007/s40259-017-0231-8 28612180 PMC5548814

[70] Elliott M Benson J Blank M Brodmerkel C Baker D Sharples KR. Ustekinumab: lessons learned from targeting interleukin-12/23p40 in immune-mediated diseases. Ann N Y Acad Sci. (2009) 1182:97–110. 10.1111/j.1749-6632.2009.05070.x 20074279

[71] Fasching P Stradner M Graninger W Dejaco C Fessler J. Therapeutic potential of targeting the Th17/Treg axis in autoimmune disorders. Molecules (2017) 22:E134. 10.3390/molecules22010134 28098832 PMC6155880

[72] Nistala K Adams S Cambrook H Ursu S Olivito B de Jager W. Th17 plasticity in human autoimmune arthritis is driven by the inflammatory environment. Proc Natl Acad Sci USA. (2010) 107:14751–6. 10.1073/pnas.1003852107 20679229 PMC2930428

[73] Cosmi L Cimaz R Maggi L Santarlasci V Capone M Borriello F. Evidence of the transient nature of the Th17 phenotype of CD4+CD161+ T cells in the synovial fluid of patients with juvenile idiopathic arthritis. Arthritis Rheum. (2011) 63:2504–15. 10.1002/art.30332 21381000

[74] Piper C Pesenacker AM Bending D Thirugnanabalan B Varsani H Wedderburn LR. T cell expression of granulocyte-macrophage colony-stimulating factor in juvenile arthritis is contingent upon Th17 plasticity. Arthritis Rheumatol. (2014) 66:1955–60. 10.1002/art.38647 24692225 PMC4190686

[75] Papp KA Griffiths CE Gordon K Lebwohl M Szapary PO Wasfi Y. Long-term safety of ustekinumab in patients with moderate-to-severe psoriasis: final results from 5 years of follow-up. Br J Dermatol. (2013) 168:844–54. 10.1111/bjd.12214 23301632

[76] Kellen R Silverberg NB Lebwohl M. Efficacy and safety of ustekinumab in adolescents. Pediatric Health Med Ther. (2016) 7:109–20. 10.2147/PHMT.S75836 29388600 PMC5683279

[77] van Vollenhoven RF Hahn BH Tsokos GC Wagner CL Lipsky P Touma Z. Efficacy and safety of ustekinumab, an IL-12 and IL-23 inhibitor, in patients with active systemic lupus erythematosus: results of a multicentre, double-blind, phase 2, randomised, controlled study. Lancet (2018) 392:1330–9. 10.1016/S0140-6736(18)32167-6 30249507

[78] Mannion ML McAllister L Cron RQ Stoll ML. Ustekinumab as a therapeutic option for children with refractory enthesitis-related arthritis. J Clin Rheumatol. (2016) 22:282–4. 10.1097/RHU.0000000000000408 27464779

[79] Kavanaugh A Puig L Gottlieb AB Ritchlin C Li S Wang Y. Maintenance of clinical efficacy and radiographic benefit through two years of ustekinumab therapy in patients with active psoriatic arthritis: results from a randomized, placebo-controlled phase III trial. Arthritis Care Res (Hoboken). (2015) 67:1739–49. 10.1002/acr.22645 26097039 PMC5063124

[80] Teng MW Bowman EP McElwee JJ Smyth MJ Casanova JL Cooper AM. IL-12 and IL-23 cytokines: from discovery to targeted therapies for immune-mediated inflammatory diseases. Nat Med. (2015) 21:719–29. 10.1038/nm.3895 26121196

[81] Montoya CL Gonzalez ML Ospina FE Tobon GJ. A rare case of amyopathic juvenile dermatomyositis associated with psoriasis successfully treated with ustekinumab. J Clin Rheumatol. (2017) 23:129–30. 10.1097/RHU.0000000000000430 28099216

[82] Weaver CT Hatton RD. Interplay between the TH17 and TReg cell lineages: a (co-)evolutionary perspective. Nat Rev Immunol. (2009) 9:883–9. 10.1038/nri2660 19935807

[83] Samson M Ghesquiere T Berthier S Bonnotte B. Ustekinumab inhibits Th1 and Th17 polarisation in a patient with giant cell arteritis. Ann Rheum Dis. (2018) 77:e6. 10.1136/annrheumdis-2017-211622 28501800

[84] Barnes MG Grom AA Thompson SD Griffin TA Pavlidis P Itert L. Subtype-specific peripheral blood gene expression profiles in recent-onset juvenile idiopathic arthritis. Arthritis Rheum. (2009) 60:2102–12. 10.1002/art.24601 19565513 PMC2782469

[85] Nistala K Varsani H Wittkowski H Vogl T Krol P Shah V. Myeloid related protein induces muscle derived inflammatory mediators in juvenile dermatomyositis. Arthritis Res Ther. (2013) 15:R131. 10.1186/ar4311 24286299 PMC3978554

[86] Ferreira RC Rainbow DB Rubio Garcia A Pekalski ML Porter L Oliveira JJ. In-depth immunophenotyping data of IL-6R on the human peripheral regulatory T cell (Treg) compartment. Data Brief (2017) 12:676–91. 10.1016/j.dib.2017.04.043 28567438 PMC5435581

[87] Bhairavabhotla R Kim YC Glass DD Escobar TM Patel MC Zahr R. Transcriptome profiling of human FoxP3+ regulatory T cells. Hum Immunol. (2016) 77:201–13. 10.1016/j.humimm.2015.12.004 26686412 PMC4761514

[88] Pesenacker AM Bending D Ursu S Wu Q Nistala K Wedderburn LR. CD161 defines the subset of FoxP3+ T cells capable of producing proinflammatory cytokines. Blood (2013) 121:2647–58. 10.1182/blood-2012-08-443473 23355538 PMC3617631

[89] Brunner HI Tzaribachev N Vega-Cornejo G Louw I Berman A Calvo Penades I. Subcutaneous abatacept in patients with polyarticular-course juvenile idiopathic arthritis: results from a phase III open-label study. Arthritis Rheumatol. (2018) 70:1144–54. 10.1002/art.40466 29481737 PMC6032847

[90] Maggi L Cimaz R Capone M Santarlasci V Rossi MC Mazzoni A. Immunosuppressive activity of abatacept on circulating T helper lymphocytes from juvenile idiopathic arthritis patients. Int Arch Allergy Immunol. (2016) 171:45–53. 10.1159/000450948 27820937

[91] Lovell DJ Ruperto N Mouy R Paz E Rubio-Perez N Silva CA. Long-term safety, efficacy, and quality of life in patients with juvenile idiopathic arthritis treated with intravenous abatacept for up to seven years. Arthritis Rheumatol. (2015) 67:2759–70. 10.1002/art.39234 26097215 PMC5054936

[92] Ruperto N Lovell DJ Quartier P Paz E Rubio-Perez N Silva CA. Abatacept in children with juvenile idiopathic arthritis: a randomised, double-blind, placebo-controlled withdrawal trial. Lancet (2008) 372:383–91. 10.1016/S0140-6736(08)60998-8 18632147

[93] Arabshahi B Silverman RA Jones OY Rider LG. Abatacept and sodium thiosulfate for treatment of recalcitrant juvenile dermatomyositis complicated by ulceration and calcinosis. J Pediatr. (2012) 160:520–2. 10.1016/j.jpeds.2011.11.057 22244459 PMC3306811

[94] Sumitomo S Nagafuchi Y Tsuchida Y Tsuchiya H Ota M Ishigaki K. A gene module associated with dysregulated TCR signaling pathways in CD4(+) T cell subsets in rheumatoid arthritis. J Autoimmun. (2018) 89:21–9. 10.1016/j.jaut.2017.11.001 29146547

[95] Orban T Beam CA Xu P Moore K Jiang Q Deng J. Reduction in CD4 central memory T-cell subset in costimulation modulator abatacept-treated patients with recent-onset type 1 diabetes is associated with slower C-peptide decline. Diabetes (2014) 63:3449–57. 10.2337/db14-0047 24834977 PMC4171657

[96] Rochman Y Yukawa M Kartashov AV Barski A. Functional characterization of human T cell hyporesponsiveness induced by CTLA4-Ig. PLoS ONE (2015) 10:e0122198. 10.1371/journal.pone.0122198 25860138 PMC4393265

[97] Szentpetery A Heffernan E Gogarty M Mellerick L McCormack J Haroon M. Abatacept reduces synovial regulatory T-cell expression in patients with psoriatic arthritis. Arthritis Res Ther. (2017) 19:158. 10.1186/s13075-017-1364-3 28679449 PMC5498994

[98] Langdon K Haleagrahara N. Regulatory T-cell dynamics with abatacept treatment in rheumatoid arthritis. Int Rev Immunol. 37:206–14. 10.1080/08830185.2018.1465943 29757670

[99] Alvarez-Quiroga C Abud-Mendoza C Doniz-Padilla L Juarez-Reyes A Monsivais-Urenda A Baranda L. CTLA-4-Ig therapy diminishes the frequency but enhances the function of Treg cells in patients with rheumatoid arthritis. J Clin Immunol. (2011) 31:588–95. 10.1007/s10875-011-9527-5 21487894

[100] Bonelli M Goschl L Bluml S Karonitsch T Hirahara K Ferner E. Abatacept (CTLA-4Ig) treatment reduces T cell apoptosis and regulatory T cell suppression in patients with rheumatoid arthritis. Rheumatology (Oxford) (2016) 55:710–20. 10.1093/rheumatology/kev403 26672908

[101] Duggleby R Danby RD Madrigal JA Saudemont A. Clinical grade regulatory CD4(+) T cells (Tregs): moving toward cellular-based immunomodulatory therapies. Front Immunol. (2018) 9:252. 10.3389/fimmu.2018.00252 29487602 PMC5816789

[102] Bluestone JA Buckner JH Fitch M Gitelman SE Gupta S Hellerstein MK. Type 1 diabetes immunotherapy using polyclonal regulatory T cells. Sci Transl Med. (2015) 7:315ra189. 10.1126/scitranslmed.aad4134 26606968 PMC4729454

[103] Tang Q Vincenti F. Transplant trials with Tregs: perils and promises. J Clin Invest. (2017) 127:2505–12. 10.1172/JCI90598 28665300 PMC5490750

[104] Brunstein CG Miller JS McKenna DH Hippen KL DeFor TE Sumstad D. Umbilical cord blood-derived T regulatory cells to prevent GVHD: kinetics, toxicity profile, and clinical effect. Blood (2016) 127:1044–51. 10.1182/blood-2015-06-653667 26563133 PMC4768428

[105] Brunstein CG Miller JS Cao Q McKenna DH Hippen KL Curtsinger J. Infusion of ex vivo expanded T regulatory cells in adults transplanted with umbilical cord blood: safety profile and detection kinetics. Blood (2011) 117:1061–70. 10.1182/blood-2010-07-293795 20952687 PMC3035067

[106] Dijke IE Hoeppli RE Ellis T Pearcey J Huang Q McMurchy AN. Discarded human thymus is a novel source of stable and long-lived therapeutic regulatory T cells. Am J Transplant. (2015) 16:58–71. 10.1111/ajt.13456 26414799

[107] Hoeppli RE MacDonald KN Leclair P Fung VCW Mojibian M Gillies J Tailoring the homing capacity of human Tregs for directed migration to sites of Th1-inflammation or intestinal regions. Am J Transplant. (2018) 19:62–76. 10.1111/ajt.14936 29766641

[108] Sicard A Boardman DA Levings MK. Taking regulatory T-cell therapy one step further. Curr Opin Organ Transplant. (2018) 23:509–15. 10.1097/MOT.0000000000000566 30063480

[109] Landman S de Oliveira VL van Erp PEJ Fasse E Bauland SCG Joosten I. Intradermal injection of low dose human regulatory T cells inhibits skin inflammation in a humanized mouse model. Sci Rep. (2018) 8:10044. 10.1038/s41598-018-28346-5 29968819 PMC6030170

[110] Spolski R Li P Leonard WJ. Biology and regulation of IL-2: from molecular mechanisms to human therapy. Nat Rev Immunol. (2018) 18:648–59. 10.1038/s41577-018-0046-y 30089912

[111] Leon K Garcia-Martinez K Carmenate T Rojas G. Combining computational and experimental biology to develop therapeutically valuable IL2 muteins. Semin Oncol. (2018) 45:95–104. 10.1053/j.seminoncol.2018.04.001 30318089

[112] Dwyer CJ Ward NC Pugliese A Malek TR. Promoting immune regulation in type 1 diabetes using low-dose interleukin-2. Curr Diab Rep. (2016) 16:46. 10.1007/s11892-016-0739-1 27076179 PMC5907489

[113] von Spee-Mayer C Siegert E Abdirama D Rose A Klaus A Alexander T Low-dose interleukin-2 selectively corrects regulatory T cell defects in patients with systemic lupus erythematosus. Ann Rheum Dis. (2015) 75:1407–15. 10.1136/annrheumdis-2015-207776 26324847

[114] Todd JA Evangelou M Cutler AJ Pekalski ML Walker NM Stevens HE. Regulatory T cell responses in participants with type 1 diabetes after a single dose of interleukin-2: a non-randomised, open label, adaptive dose-finding trial. PLoS Med. (2016) 13:e1002139. 10.1371/journal.pmed.1002139 27727279 PMC5058548

[115] Rosenzwajg M Churlaud G Mallone R Six A Derian N Chaara W. Low-dose interleukin-2 fosters a dose-dependent regulatory T cell tuned milieu in T1D patients. J Autoimmun. (2015) 58:48–58. 10.1016/j.jaut.2015.01.001 25634360 PMC8153751

[116] Matsuoka K Koreth J Kim HT Bascug G McDonough S Kawano Y. Low-dose interleukin-2 therapy restores regulatory T cell homeostasis in patients with chronic graft-versus-host disease. Sci Transl Med. (2013) 5:179ra143. 10.1126/scitranslmed.3005265 23552371 PMC3686517

[117] Koreth J Kim HT Jones KT Lange PB Reynolds CG Chammas MJ. Efficacy, durability, and response predictors of low-dose interleukin-2 therapy for chronic graft-versus-host disease. Blood (2016) 128:130–7. 10.1182/blood-2016-02-702852 27073224 PMC4937358

[118] Trotta E Bessette PH Silveria SL Ely LK Jude KM Le DT. A human anti-IL-2 antibody that potentiates regulatory T cells by a structure-based mechanism. Nat Med. (2018) 24:1005–14. 10.1038/s41591-018-0070-2 29942088 PMC6398608

[119] Lee SY Cho ML Oh HJ Ryu JG Park MJ Jhun JY. Interleukin-2/anti-interleukin-2 monoclonal antibody immune complex suppresses collagen-induced arthritis in mice by fortifying interleukin-2/STAT5 signalling pathways. Immunology (2012) 137:305–16. 10.1111/imm.12008 23167249 PMC3530086

[120] Yokoyama Y Iwasaki T Kitano S Satake A Nomura S Furukawa T. IL-2-Anti-IL-2 monoclonal antibody immune complexes inhibit collagen-induced arthritis by augmenting regulatory T cell functions. J Immunol. (2018) 201:1899–906. 10.4049/jimmunol.1701502 30143591

[121] Zeng Z Yu K Chen L Li W Xiao H Huang Z. Interleukin-2/Anti-Interleukin-2 immune complex attenuates cardiac remodeling after myocardial infarction through expansion of regulatory T cells. J Immunol Res. (2016) 2016:8493767. 10.1155/2016/8493767 27144181 PMC4837274

[122] Spangler JB Trotta E Tomala J Peck A Young TA Savvides CS. Engineering a single-agent cytokine/antibody fusion that selectively expands regulatory T cells for autoimmune disease therapy. J Immunol. (2018) 201:2094–106. 10.4049/jimmunol.1800578 30104245 PMC6173196

[123] Bell CJ Sun Y Nowak UM Clark J Howlett S Pekalski ML. Sustained in vivo signaling by long-lived IL-2 induces prolonged increases of regulatory T cells. J Autoimmun. (2015) 56:66–80. 10.1016/j.jaut.2014.10.002 25457307 PMC4298360

[124] Ward NC Yu A Moro A Ban Y Chen X Hsiung S. IL-2/CD25: a long-acting fusion protein that promotes immune tolerance by selectively targeting the IL-2 receptor on regulatory T cells. J Immunol. (2018) 201:2579–92. 10.4049/jimmunol.1800907 30282751 PMC6200646

[125] Kunz DJ Gomes T James KR. Immune cell dynamics unfolded by single-cell technologies. Front Immunol. (2018) 9:1435. 10.3389/fimmu.2018.01435 29997618 PMC6028612

[126] Goltsev Y Samusik N Kennedy-Darling J Bhate S Hale M Vazquez G. Deep profiling of mouse splenic architecture with CODEX multiplexed imaging. Cell (2018) 174, 968–81 e915. 10.1016/j.cell.2018.07.010 30078711 PMC6086938

[127] Dixon AR Bathany C Tsuei M White J Barald KF Takayama S. Recent developments in multiplexing techniques for immunohistochemistry. Expert Rev Mol Diagn. (2015) 15:1171–86. 10.1586/14737159.2015.1069182 26289603 PMC4810438

[128] Ehlers MR Nepom GT. Immune-directed therapy for type 1 diabetes at the clinical level: the Immune Tolerance Network (ITN) experience. Rev Diabet Stud. (2012) 9:359–71. 10.1900/RDS.2012.9.359 23804273 PMC3740703

